# Mechanism of G1 arrest in the *Drosophila *eye imaginal disc

**DOI:** 10.1186/1471-213X-7-13

**Published:** 2007-03-02

**Authors:** Luis M Escudero, Matthew Freeman

**Affiliations:** 1 MRC Laboratory of Molecular Biology, Hills Road, Cambridge CB2 0QH, UK

## Abstract

**Background:**

Most differentiating cells are arrested in G1-phase of the cell cycle and this proliferative quiescence appears important to allow differentiation programmes to be executed. An example occurs in the *Drosophila *eye imaginal disc, where all cells are synchronized and arrested in G1 phase prior to making a fate choice either to initiate the first round of photoreceptor differentiation or to re-enter one terminal mitosis.

**Results:**

We have analysed the mechanism of this temporally regulated G1-phase in order to develop an integrated model of this proliferative regulation. We find that an overlapping set of cell cycle inhibitors combine to form an efficient barrier to cell cycle progression. This barrier depends on both the primary secreted signals that drive retinal development, Dpp and Hh. Each of these has distinct, as well as partially overlapping functions, in ensuring that Cyclin E and dE2F1 are kept in check. Additionally, inhibition of Cyclin A by Roughex is essential, and this regulation is independent of Dpp and Hh.

**Conclusion:**

One implication of these results is to further support the idea that Cyclin A has important functions in S-phase entry as well as in mitosis. The unexpectedly complex network of regulation may reflect the importance of cells being uniformly ready to respond to the inductive signals that coordinate retinal differentiation.

## Background

All eukaryotes use the same fundamental machinery to drive cell cycle progression but multicellular organisms face the additional challenge of regulating the time and place of proliferation throughout development. A well conserved aspect of this developmental regulation is that cells normally arrest in G1-phase of the cell cycle prior to differentiation. This provides a quiescent stage for differentiation to begin and, once cells start to adopt their terminal fate, most will never re-enter a proliferative state. In tumorigenesis, however, re-entry of arrested cells into abnormal proliferation can occur [1].

The *Drosophila *eye provides an experimentally amenable example of developmentally regulated proliferation and has therefore been extensively studied as a model. Differentiation of the individual facets (ommatidia) of the compound eye occurs in a moving wave from the posterior to the anterior of the eye imaginal disc, implying that there is a gradient of increasing cell maturity from anterior to posterior in the disc. The front of the wave of development is marked by an indentation known as the morphogenetic furrow (MF) [2]. Several hours prior to the passage of the MF (and therefore anterior to it) all cells in the eye disc arrest in G1-phase [3,4]. After the MF passes, those cells that have not yet started to differentiate as photoreceptors re-enter S-phase for one terminal round of division, the "second mitotic wave" [5].

Here we focus on the mechanisms responsible for establishing and maintaining G1-arrest of cells prior to the MF (in what we call non-proliferative region, NPR). Specifically, we aim to understand how cells become synchronous and arrest in G1-phase and to learn how the signals that drive the wave of eye development direct this process. This aspect of developmental control of proliferation in *Drosophila *is not well understood, although it has been investigated in a number of different contexts including the eye, the wing and the embryo [4,6-12]. Most significantly in the eye, Decapentaplegic (Dpp, the *Drosophila *homologue of BMP ligands) signalling is required to maintain G1 arrest in the anterior region of the arrested zone. Dpp appears to repress Cyclin E, since the removal of several CycE inhibitors [7,13,14] causes a similar phenotype to the lack to the Dpp receptor, *thick veins *(*tkv*) or to the overexpression of a CycE transgene [12]. More recently, Firth and Baker have concluded that Dpp and Hedgehog (Hh) act redundantly, and between them are sufficient to arrest cells in G1 [7]. This involvement of Dpp and Hh is consistent with their roles as the primary secreted signals that drive MF progression. Finally, the overexpression of Cyclin A or the absence of the cyclin kinase inhibitor Roughex (Rux) can also induce S-phase entry in the NPR [4,15]. Together, these earlier studies have identified a number of different mechanisms of control of G1 arrest but it is notable that no common checkpoint has been identified and no unified view of the process has yet been proposed.

Providing an explanation for this lack of a clear model, our results indicate that in the eye disc no single component is fully responsible for the developmental cell cycle arrest prior to the morphogenetic furrow. We show that first, Hh and Dpp together promote entry into mitosis of the cells prior to G1 arrest, thereby driving cells into G1-phase and initiating the NPR. Then, an overlapping set of cell cycle inhibitors combine to form an efficient and robust barrier to cell cycle progression. This barrier depends partially on the inhibition of Cyclin E and dE2F1 activity, also under the control of Dpp and Hh. However, in contrast to previous work [7], our results show that the role of Hh and Dpp in maintaining G1 arrest, is largely confined to the anterior part of the NPR. The inhibition of Cyclin A by Rux becomes the major barrier to S-phase entry in the posterior region and, significantly, this is independent of both Dpp and Hh. This analysis of the relative importance of the different players involved in G1 arrest allows us to integrate them into an overall model of signal-regulated synchronisation and proliferative arrest.

## Results

### Defining the region of G1 arrest in the eye imaginal disc

Between the proliferating cells in the anterior of the eye imaginal disc and the SMW is a broad band of cells that are arrested in G1-phase [3,16]; they span about 11–14 rows of cells. Given the accepted view that the morphogenetic furrow moves forward, on average, by one row of ommatidia (3–4 cell rows) every 70 min [17], we estimate that on average cells remain in this G1 arrest for five to six hours. Interestingly, this is only slightly longer than the estimate of the normal G1 phase of cells in the proliferating region of the disc. This is based on the observation that the doubling time for cells in the eye disc is approximately 12 hours [18], and that the proportion of cells in G1 phase in proliferating disc cells is one third [19]. These estimates imply that the formation of this non-proliferative region (NPR) depends significantly on cell cycle synchronization as they approach the MF, as well as on specific arrest mechanisms.

**Figure 1:**
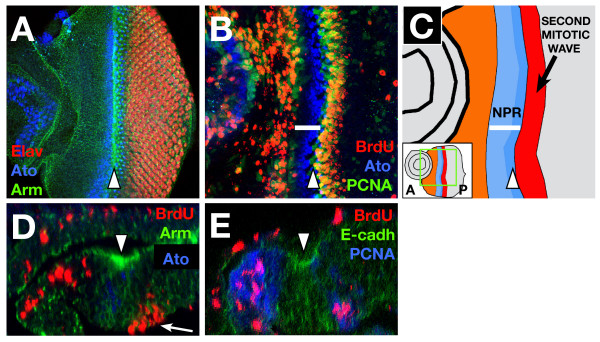
**Pattern of proliferation and G1 arrest in the eye**. (A) Projection of several confocal sections of a WT eye disc showing Armadillo (green), Elav (red) and Ato (blue) localization. The morphogenetic furrow (MF, arrowhead in all figures) is highlighted by accumulation of Armadillo (β-catenin), which outlines cell membranes. Ato expression appears between 6–8 cells anterior to the MF and becomes limited to the R8 photoreceptor just posterior to the furrow. Elav is expressed in all the photoreceptor cells posterior to the Ato expressing R8. In this and all subsequent figures, anterior is to the left. (B) WT eye disc showing the incorporation of BrdU (red) and the expression of *PCNA-GFP *reporter (indicating dE2F1 activation, green) and Ato protein (blue). Anterior to the furrow there is a non-proliferative region, without BrdU incorporation (white bar). The pattern of *PCNA-GFP *expression coincides with the regions of proliferation. (C) Scheme showing the different proliferating regions of the eye disc. The panel shows a drawing of the disc in 1B. The inset shows a picture of the whole disc. From anterior to posterior: orange marks the region of undifferentiated cells that proliferate randomly; the NPR is marked in blue (its extent is marked by the white bar), with the darker zone showing the morphogenetic furrow (also marked by an arrowhead); finally, the red band marks the region of the second mitotic wave. (D) Z-axis reconstruction of a WT eye disc. The outlines of the cells are shown by Armadillo (Arm) expression (green). The peripodial membrane appears at the top with some BrdU positive cells (red). In the disc proper there is a high accumulation of Arm protein in the apical part of the MF (arrowhead). S-phase nuclei in the SMW are basally located (arrow), whereas anterior to the NPR, BrdU positive nuclei are more apical. The NPR includes the furrow cells (that accumulate high apical levels of Arm) and between 4–6 rows of more anterior cells. Ato expressing cells (blue) are restricted to the disc proper. (E) The cytoplasmic expression of *PCNA-GFP *reporter (blue) is seen in cells that are completing the cell cycle in the most anterior part of the NPR, but is not expressed in the cells of the MF (that accumulate E-cadherin in the apical region, in green). In the SMW the cells with BrdU positive nuclei also express the *PCNA-GFP*.

In this paper we define the NPR as corresponding to the absence of BrdU incorporation anterior to the SMW (Fig. 1A–C). So, from anterior to posterior in the eye disc are 1) the region where undifferentiated cells proliferate randomly; 2) the NPR (which includes the morphogenetic furrow); and 3) the second mitotic wave (Fig. 1C). Note that in the anterior part of the NPR, cells do not enter G1 arrest until after mitosis, so the last cells to be detected by BrdU staining actually enter G1 one or two rows later, after completion of G2 and M-phases. The activity of dE2F1 (a homologue of mammalian E2F, a transcription factor responsible for the expression of many S-phase components [20]) is largely absent from the NPR, as measured by the expression of a *PCNA-GFP *reporter [21], which contains the dE2F1 binding sequence from the promoter of the PCNA gene (Figure 1B, E). We observed that the downregulation of BrdU staining coincides precisely with the onset of Atonal (Ato) expression [22,23] anterior to the furrow (Fig. 1A, B, D), and with the loss of Homothorax (Hth) expression (not shown). However, in clones of *ato *or *hth *mutant cells enter G1 arrest normally, implying that these genes are not essential for establishing the NPR.

To characterise the anatomy of the NPR further, we have used 3-dimensional reconstruction software to image the disc (Fig. 1D, E). This clearly showed that the morphogenetic furrow forms the posterior region of the NPR. It also allowed a clear view of the 'peripodial membrane', the layer of squamous cells that overlie the disc-proper and which can influence aspects of disc development [24]. BrdU positive cells are present in the peripodial membrane, but these were randomly distributed, indicating the absence of coordination of G1 arrest between the two adjacent epithelia. To prevent the BrdU positive cells in the peripodial membrane being misinterpreted as disc proper cells, in all subsequent confocal projections we used Ato expression as a reference for disc proper.

### The NPR requires repression of Cyclin E, dE2F1 and Cyclin A

**Figure 2:**
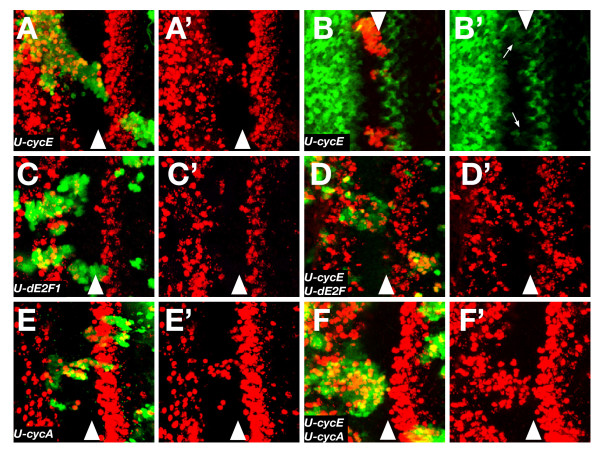
**Effect of overexpression of cyclins in the NPR**. All panels except B show BrdU staining (red) of eye disc harbouring different overexpression clones marked with GFP (green). (A) The overexpression of CycE is able to induce ectopic BrdU incorporation with a high efficiency in the anterior part of the NPR, but less so the posterior, where only a few cells enter S phase. (B) The *PCNA-GFP *reporter (green) is active (arrows) in the CycE overexpression clones (marked by presence of β-gal in red) within the NPR. (C) In dE2F1 overexpression clones there is BrdU incorporation only in the anterior part of the NPR. (D) The overexpression of CycE and dE2F1 can induce incorporation of BrdU in anterior and posterior cells of the NPR. (E) CycA overexpressing cells incorporate BrdU in any part of the NPR, but with a higher efficiency in the posterior part, where most cells are BrdU positive. (F) Simultaneous overexpression of CycE and CycA induces a high proportion of BrdU incorporation in any part of the NPR.

In *Drosophila*, the main activators of S-phase entry are Cyclin E [25], dE2F1 [20,26] and Cyclin A [7,9,15,27]. All are inactive in the NPR and the protein levels of Cyclins E and A are very low in this region [4,12]. To examine the relative importance of the absence of these S-phase triggers in the maintenance of G1 arrest, we overexpressed them using the flip-out Gal4 technique [28]. This technique permits the direct comparison between overexpressing cells and neighbouring wild-type cells, thereby providing a more detailed view of the phenotype than is possible with simple Gal4 misexpression. The ectopic expression of Cyclin E induced BrdU incorporation very efficiently in the anterior region of the NPR (Fig. 2A and [12]). More rarely, we observed a few cells in S-phase in more posterior regions of the NPR. Cyclin E regulates dE2F1 activity by inhibiting Rbf [29], the *Drosophila *homologue of the retinoblastoma factor. Consistent with this, cells overexpressing Cyclin E expressed the dE2F1 reporter construct, *PCNA-GFP *(Fig. 2B).

Overexpression of *UAS-dE2F1 *(combined with its obligate partner *UAS-Dp*) [30] caused a weaker phenotype than Cyclin E, although the pattern was similar: in this case, occasional BrdU positive cells were seen but only in the anterior region of the NPR (Fig. 2C). This result is consistent with the loss of function phenotype of *rbf*, the main inhibitor dE2F1 activity, which causes ectopic S-phase cells only in the most anterior regions of the NPR [13]. Taken together, the overexpression of Cyclin E and dE2F1/Dp in the NPR implies that at least one additional mechanism must promote G1 arrest in the posterior cells of the NPR; unlike the anterior cells, they are refractory to overexpression of Cyclin E or dE2F1/Dp. Note that because of the progressive development of the retina, the anterior cells mature into the posterior cells, so this different responsiveness to Cyclin E and dE2F1/Dp actually represents a developmental difference rather than a spatial one.

The co-overexpression of *UAS-cycE *and *UAS-dE2F1/Dp *produced a slightly stronger phenotype than *UAS-cycE *overexpression alone (in which endogenous dE2F1 is also activated): S-phase is efficiently activated in the anterior but less so in the posterior part of the NPR (Fig. 2D). This result is consistent with the earlier report that in clones mutant for both *rbf *and *dap*, inhibitors of dE2F1 and Cyclin E, respectively, the cells in the whole of the NPR do not arrest [7]; in our hands these double mutant *rbf dap *clones can only sometimes induce ectopic BrdU staining in the anterior region of the NPR and have even weaker effects in the posterior region (not shown). We take the difference between the more powerful effect of ectopic Cyclin E and dE2F1, and the weaker effect of *rbf dap *to indicate that overexpression of activators is a more powerful trigger than removal of natural repressors.

Cyclin A has a less well defined role in S-phase activation but the evidence for its participation is nevertheless strong [7,15,27]. Interestingly, its overexpression in the NPR gave a different result to Cyclin E or dE2F1. *UAS-cycA *induced ectopic BrdU staining in the whole width of the NPR, but was most efficient in the most posterior domain, where Cyclin E and dE2F1 are less effective (Fig. 2E and [15]). The combination of both transgenes, *UAS-cycA *and *UAS-cycE *strongly induced ectopic S-phase entry in cells throughout the NPR: almost all overexpressing cells failed to arrest in G1 (Fig. 2F).

Overall, our data provide strong evidence that G1 arrest in the NPR is controlled by multiple mechanisms with distinct regional effects. Specifically, in the anterior region of the NPR, which corresponds to the early stages of G1 arrest, the repression of Cyclin E and dE2F1 activity is critically important, while in the posterior half, where the cells are more mature, the repression of Cyclin A becomes necessary for the maintenance of these cells in the G1 phase.

### Control of G1 arrest by Hh and Dpp

The NPR is the earliest visible change in cells as they enter the wave of development that will eventually produce the adult retina. Ultimately, the whole wave of eye development is propagated by secreted Dpp and Hh signals. Consistent with this, it has previously been reported that Dpp is responsible for the G1-arrest in the anterior part of the NPR [8,11]; most recently, Firth and Baker have reported that Dpp and Hh act redundantly to induce the whole NPR [7]. To understand the mechanism in detail by which Dpp and Hh cause cells to exit from proliferation and arrest/synchronise in G1, we genetically blocked the two pathways, either alone or together. We used mutations in Thick Veins (Tkv), the Dpp receptor; Smoothened (Smo), the membrane associated transducer of Hh signalling; Mad, an essential intracellular transducer of Dpp signalling; and Ci, a nuclear effector of Hh signalling. Note that Ci has constitutive repressor activity as well as being a Hh-dependent activator [31], implying that loss of Ci is not always equivalent to loss of Smo. For this reason, simultaneous loss of Smo and Tkv represents the best way of analysing the loss-of-function phenotypes of the Hh and Dpp pathways in the eye disc [32,33].

#### Initial synchronisation of cells as they enter the NPR

**Figure 3:**
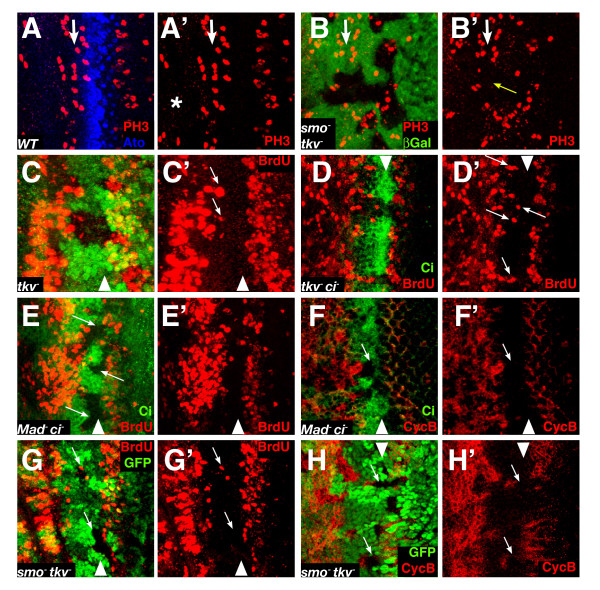
**Dpp and Hh control G1 arrest primarily in the anterior of the NPR**. (A) WT eye disc showing the cells in mitosis as marked by phosphohistone H3 antibody (PH3, in red) and the Ato pattern of expression (blue). The white arrow marks the line of synchronized mitosis anterior to the NPR in A and B. In the region anterior to this line the frequency of phosphohistone H3 positive cells is very low (asterisk) (B) Eye disc with a large *smo*^3 ^*tkv*^*a*12 ^*M*^+ ^clone (absence of green). The yellow arrow marks the region where the distinct line of mitosis (pH3, red; the so-called first mitotic wave) is lost. (C) *tkv*^*a*12 ^clones (absence of green) showing some BrdU positive cells (red) in the anterior part but not in the posterior (arrows). (D) Similarly, in *tkv*^*a*12 ^*ci*^94 ^clones (marked by the absence of Ci antibody in green) there are no BrdU positive cells in the posterior region of the NPR (red). The arrows mark some BrdU cells in the anterior part of the NPR. (E-F) *Mad*^12 ^*ci*^94 ^clones (marked by absence of Ci, green) stained for BrdU (E) and CycB (F) in red. The arrows mark clones in the posterior part of the NPR where there is no ectopic BrdU incorporation or CycB accumulation. (G) *smo*^3 ^*tkv*^*a*12 ^clones (arrows) marked by absence of β-gal, in green (arrows). The picture shows a representative clone in the lower part of the panel with no ectopic BrdU positive cells (red) (H) Ectopic CycB (red) accumulates only in the anterior region of the NPR in *smo*^3 ^*tkv*^*a*12 ^clones (absence of green). This is highlighted in clones that span the whole NPR, where there is clear CycB accumulation that does not reach the most posterior cells (arrows).

One of the earliest signs of the NPR is an increase in the number of mitotic cells immediately prior to the G1 arrest region. This is seen in wild type discs as a well defined line of phosphohistone H3 (pH3) positive cells immediately anterior to the NPR (Fig. 3A), and contrasts with the much more sparsely scattered pH3-positive cells further anterior. We observed that in the *smo tkv *double mutant clones (but not in the *tkv *or *smo *clones alone, not shown) this clear alignment of mitotic cells was lost (Fig. 3B), implying that cells were no longer being efficiently driven into mitosis. Note that pH3 only stains cells in for a short period of the cell cycle, so its absence cannot be taken as a sign of proliferative arrest. Instead we interpret this result to indicate a function for Hh and Dpp in accelerating through mitosis those cells that are already 'pre-mitotic' or in later stages of the cell cycle.

#### A differential requirement for Dpp and Hh signalling in the posterior and anterior of the NPR

Loss of *smo *alone causes a slight delay in entry to the NPR, by one or two rows of cells, but no ectopic BrdU staining occurs in the rest of the region [7]. We examined clones of *tkv *as well as double mutant clones for *Mad ci*, or *tkv ci*, or *smo tkv *(Fig. 3C–H). All four mutant conditions caused ectopic BrdU incorporation and Cyclin B expression in the anterior region of the NPR but none caused significant ectopic S-phase induction in the posterior region (this is especially clear in the case of Cyclin B staining (Fig. 3F, 3H). Of these four conditions, *smo tkv *was the only one in which occasional BrdU positive cells were detected in the posterior region (6 out of 32 clones; no more than 2–3 positive cells in any clone). Three points emerge from these data. First, they imply a clear difference between the maintenance of G1 in the anterior and posterior of the NPR. Second, Firth and Baker reported ectopic BrdU incorporation and Cyclin B accumulation in *Mad ci *clones in the whole NPR, not just the anterior region. We used the same alleles as they did but did not observe any posterior BrdU staining. Third, there is no evidence for a requirement for Ci in this process: *tkv ci *clones are indistinguishable from clones of *tkv *alone.

#### Redundancy of Smo and Tkv in the regulation of dE2F1 activity

**Figure 4:**
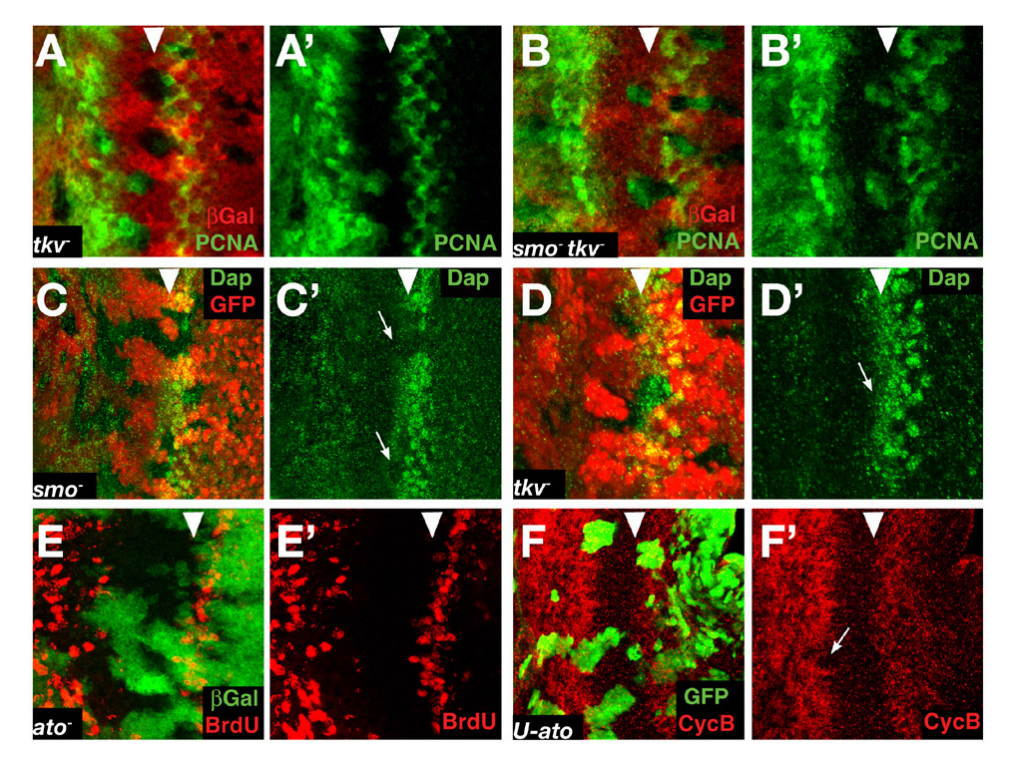
**Factors contributing to G1 arrest**. (A-B) *PCNA-GFP *expression (green) in discs with *tkv*^*a*12 ^(A) and *smo*^3 ^*tkv*^*a*12 ^(B) clones. Upregulation in the posterior part of the NPR only occurs in the *smo*^3 ^*tkv*^*a*12 ^clones. (C-D) Eye disc with *smo*^3 ^(C) and *tkv*^*a*12 ^(D) clones (absence of GFP in red), stained for Dap antibody in green. The loss of *smo*, but not *tkv*, causes the loss of Dap in the furrow (arrows). (E) Eye disc with *ato*^1 ^*M*^+ ^clones (absence of green) showing no ectopic BrdU incorporation (red) in the NPR. (F) *UAS-ato *overexpression clones (marked by GFP in green) can inhibit CycB accumulation (red) close to the anterior part of the NPR (arrow in F').

Loss of *tkv *and *smo *together caused ectopic dE2F1 activity (as assayed by *PCNA-GFP *expression) in the whole NPR (Fig. 4B), while loss of *tkv *alone activated dE2F1 in the anterior region alone (Fig. 4A); loss of *smo *alone had no effect on dE2F1 activity (not shown). Therefore both signals contribute, in a partially redundant manner, to the normal downregulation of dE2F1 activity in the NPR. Note that this activation of dE2F1 is not sufficient to induce ectopic S-phase entry in the posterior region of the NPR (recall that there was almost no ectopic BrdU or Cyclin B accumulation in posterior *smo tkv *clones), which is consistent with our observation above that ectopic dE2F1 is also not sufficient to trigger S-phase in this same region.

#### A non-redundant requirement for Hh signalling in the expression of Dacapo

Loss of *smo *alone abolished the expression of Dacapo (Dap), an inhibitor of CycE activity [34,35]. Careful examination showed that Dap expression begins in the posterior domain of the NPR and remains detectable through to the posterior of the SMW (Fig. 4C, 4D). We observed that Dap expression disappears in the *smo*^3 ^clones, but not in the *tkv*^*a*12 ^clones or *ci*^94 ^clones (Fig. 4C, 4D and not shown). This implies that Dap expression is not dependent on Dpp signalling. The result with *ci *clones implies that, consistent with earlier reports [36,37], there is no positive role for Ci in Hh signalling in this context. We also observed Dap down regulation when the constitutive Ci repressor form, *UAS-ci*^*CELL *^was overexpressed in clones (not shown). We therefore infer that Hh signalling is needed to remove the Ci repressor form in the NPR, rather than directly activating Dap expression.

These genetic experiments with the Hh and Dpp pathways point to several substantial conclusions. 1) Both pathways are required to promote the last coordinated mitosis previous to the G1 arrest. 2) G1 arrest in the anterior region of the NPR depends primarily on Dpp signalling. 3) More generally, G1 arrest is regulated differentially in the anterior and posterior of the NPR; in neither region is Ci-dependent activation required. 4) There is a redundancy between Hh and Dpp in the control of dE2F1 activity in the posterior of the NPR. 5) There is a non-redundant requirement for Hh to activate Dap expression (and thereby inhibit Cyclin E) in the posterior of the NPR; this activation requires Smo but, again, not Ci. Together, these data imply that while there is some redundancy between Hh and Dpp signalling in the control of the NPR, non-overlapping functions can also be identified. They also imply that, contrary to an earlier report [7], Hh and Dpp signalling do not comprise the whole mechanism of G1 arrest: in the posterior region of the NPR, some other factor must prevent cells from S-phase entry, even when Hh and Dpp signalling are completely blocked.

### A secondary role for Atonal in maintaining G1 arrest?

Ato expression in the NPR is induced by both Hh and Dpp pathways [32]. Together with the involvement of other proneural genes in G1 arrest in the wing margin [10], this suggests a possible role of this gene in the establishment of the NPR. However, we did not observe ectopic BrdU positive cells in Ato loss-of-function clones encompassing the NPR (Fig. 4E). On the other hand, about 55% of Atonal overexpression clones showed a clear precocious entry into the NPR (Fig. 4F), suggesting that Atonal may influence mitotic progression prior in the NPR.

### Rux is necessary for the G1 arrest in the NPR

**Figure 5:**
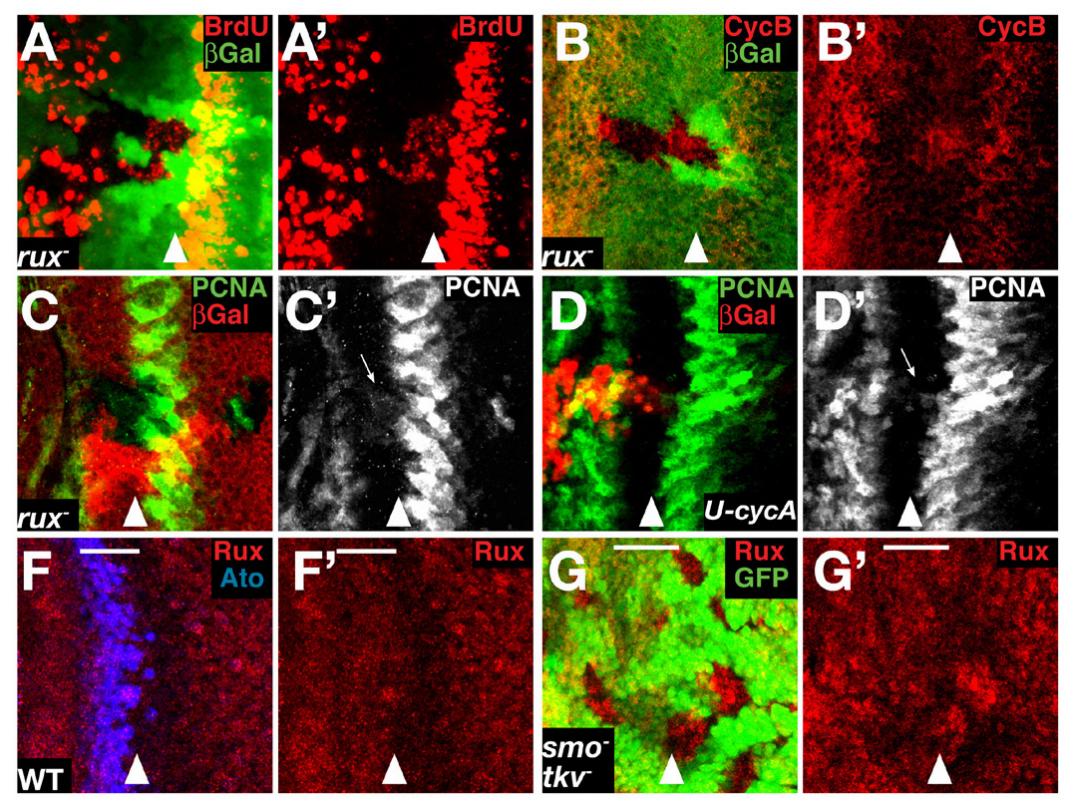
**Rux is necessary to maintain G1 arrest in the posterior of the NPR**. (A-B) *rux*^8 ^clones (absence of β-galactosidase in green) stained for BrdU (A) and CycB (B) in red. Both ectopic BrdU incorporation and CycB accumulation lie predominantly in the posterior part of the NPR, although occasional BrdU positive cells are also seen more anteriorly. (C-D) *PCNA-GFP *reporter expression (green in C and D, and white in C' and D') in discs with *rux*^8 ^clones (absence of red in C) and CycA overexpression clones (marked by presence of β-galactosidase (red in D). The *PCNA-GFP *reporter is slightly activated in the *rux *clone in the NPR (arrow in C') and in cells overexpressing CycA in the NPR (arrow in D'). (F-G) *rux-lacZ *transgene expression (red) in a WT disc (F) and in a disc harbouring *smo*^3 ^*tkv*^*a*12 ^double mutant clones. The transgene is expressed in the NPR, the position of which is localised by the expression of Ato (blue in F), and its levels decreases just posterior to the MF. The levels of *rux-lacZ *increase in the double mutant cells for *smo *and *tkv *(marked by absence of green in G). The bar indicates the width of the NPR.

The observation that Cyclin A overexpression induced BrdU incorporation in the NPR suggested that Cyclin A inhibition is important for the arrest of these cells. We therefore examined the phenotype of removing Roughex (Rux), a cytoplasmic inhibitor of Cyclin A activity [4,15,38,39]. *rux *mutant eye discs have increased proliferation anterior and posterior to the morphogenetic furrow [4]. To dissect this phenotype precisely, we induced loss of function clones of the null allele *rux*^8^. In distinction to the loss of *smo *and *tkv*, cells in the most posterior part of the NPR are no longer arrested in G1, demonstrated by the high number of cells that incorporate BrdU and the accumulation of Cyclin B (Fig. 5A–B); anterior cells are affected only slightly and many remain arrested in G1 (Fig. 5A). This phenotype is similar to that obtained by overexpressing Cyclin A and supports the idea that Rux represses Cyclin A activity and plays a significant role in maintaining G1 arrest, particularly in the posterior of the NPR.

*rux*^8 ^clones also displayed slight ectopic activation of the *PCNA-GFP *transgene in the NPR (Fig. 5C) suggesting that the powerful activation of S-phase in these clones might rely on more than just Cyclin A activation. However, since Rux is reported to be a specific regulator of Cyclin A, and is therefore not expected to affect dE2F1 activity directly, we tested whether this upregulation of dE2F1 was triggered by Cyclin A activity. Indeed, Cyclin A overexpression was also able to induce weakly the PCNA reporter in the NPR (Fig. 5D), suggesting that the slight gain of dE2F1 activity in *rux *clones was indirect.

Rux antibodies are not sensitive enough to detect the endogenous protein by immunofluorescence, so the expression pattern of *rux *in the eye disc was analysed using a *rux-lacZ *transgene that is able to rescue the *rux *phenotype and is therefore thought to represent faithfully the expression of the gene [15]. *rux-lacZ *is expressed generally in the eye disc, including in the NPR, with a decrease just posterior to the morphogenetic furrow as the second mitotic wave initiates (Fig. 5F). Expression of *rux *was not dependent on Dpp or Hh signalling: *rux-lacZ *was not down-regulated in *smo tkv *double mutant clones. On the contrary, *rux-lacZ *was elevated, indicating that Hh and Dpp normally repress *rux *expression in the second mitotic wave (Fig. 5G). This result is consistent with our other data that imply that Hh and Dpp are important determinants of G1 arrest only in the anterior domain of the NPR. In the posterior domain, when the cells have developed further, Cyclin A and Rux become the key regulators of G1 arrest.

## Discussion

### Mitotic synchrony vs. G1 Arrest

In this paper we have focused on the mechanism of G1 arrest in the eye imaginal disc, the first overt sign of retinal differentiation. Although the NPR has sometimes been considered to be merely the beginning of the morphogenetic furrow, our 3D image analysis clearly shows that it initiates well before the cells alter their shape. Just anterior to the cells arrested in G1, there is a increased number of mitotic cells suggesting a coordinated activation of their entry into mitosis. This could represent the subset of cells that are in S or G2 phases and that are accelerated through the cell cycle in order to become arrested in G1. We have shown that Hh and Dpp both have a novel role in this first mitotic synchronisation. This could be mediated by String (the homologue of Cdc25 phosphatase in *Drosophila*) since its RNA is upregulated in these cells [4]. Obviously, not all cells will be poised to enter mitosis immediately upon receiving these signals but we imagine that their function is to drive cells through mitosis with as little delay as possible.

Based on several lines of published evidence and our data [17-19], we estimate that cells in the NPR spend only a little more time in G1 phase than the proliferating anterior cells of the eye disc (see above). Coupled with the sharp onset of G1-arrest, this implies that the NPR is at least partly a consequence of cells being triggered to enter G1 synchronously. However, the observation that the loss of *tkv *causes ectopic S-phase entry in the anterior part of the NPR, and the fact that G1-phase is extended, albeit not greatly, both indicate the existence of a mechanism for preventing cells precociously entering S-phase in the NPR. In other words, we conclude that the NPR is a consequence of a combination of cell cycle synchronisation and specific arrest mechanisms.

### Dpp and Hh make distinct contributions to G1 arrest

The data presented here, coupled with several previous reports, point to the control of the NPR being dependent on multiple inputs. The earliest stage we observe is the Hh and Dpp-dependent wave of mitosis that immediately precedes the NPR; this synchronises cells at the start of the NPR. Immediately afterwards, the trigger for cells to not re-enter S-phase is primarily Dpp, which is secreted by cells immediately posterior to the MF and diffuses anteriorly to exert its effects [8,11]. We see little evidence for substantial Hh involvement in this G1 initiation step. Later in development, and therefore more posteriorly in the NPR, there is again more pronounced redundancy between Dpp and Hh, in the regulation of dE2F1 activity. *tkv *mutant cells show ectopic dE2F1 activity in the anterior region of the NPR but not in the posterior region. Cells that are mutant for both *tkv *and *smo*, however, activate dE2F1 across the whole NPR, indicating that in the posterior region, a combination of Dpp and Hh signalling contributes to G1 arrest. The distinction between the regulation of G1 arrest in anterior and posterior regions of the NPR, an indication that as cells mature their requirements change, is further emphasised by the non-redundant posterior requirement of Smo for the expression of Dacapo, a Cyclin E inhibitor. This implies that Hh signalling from the cells posterior to the furrow contributes to G1 arrest in the final stages of the NPR, although loss of this signalling does not induce S-phase. This illustrates a major theme of our work, that G1 arrest in the NPR is maintained by multiple mechanisms.

Overall, one of the main conclusions we draw from these data is that, together, Dpp and Hh exert precise regulation of two S-phase activators, Cyclin E and dE2F1, both of which must be kept in check in the NPR.

Our data also suggest that Atonal (which is activated in the NPR by Hh and Dpp) might play a secondary role in the onset of the NPR. Although loss of *ato *alone does not affect the NPR, indicating that the G1 arrest induced by Hh, Dpp and Rux does not absolutely require Atonal function, we found that the overexpression of *ato *just anterior to the NPR, can sometimes accelerate G1 entry. Interestingly, this is similar to the phenotype of *ci *LOF clones [7] in which there is this precocious entry into G1. As *ci *clones show an upregulation of Ato (our unpublished results), this provides a possible explanation of the earlier result.

The core cell cycle components that we have analysed are of course widely conserved, as are their functions. In this paper we have focused on the precise regulation of G1 arrest in one tissue – the eye imaginal disc – where Hh and Dpp induce development. Comparing our data with what has been shown in other systems, we conclude that different signalling pathways are used to regulate the cell cycle machinery in different contexts. The important point is that a precise and highly regulated set of developmental signals are used to ensure proper control of G1 and S-phase entry.

### Repression of Cyclin A is necessary for G1 arrest

The central role of Dpp and Hh signalling in initiating mitotic synchrony and maintaining G1 arrest in the NPR is consistent with the primary and exclusive role these signals have in driving the progress of eye development. It was therefore a surprise to find that blocking Dpp and Hh reception was not sufficient to trigger S-phase efficiently in posterior cells of the NPR. This particular conclusion contradicts that of Firth and Baker [7], who ascribed G1 arrest fully to Dpp and Hh-dependent processes. We do not know the cause of this disagreement but note that we have included their mutant stock in our study and obtained a different result from that which they report. In addition, our results are consistent with earlier data that de-repression of Cyclin E and dE2F1 is not sufficient to induce S-phase entry in the posterior of the NPR: for example, ectopic BrdU incorporation was seen in anterior regions of the NPR in clones of cells mutant for the Cyclin E inhibitors *dap *and *archipelago*, and the dE2F1 inhibitor, *rbf*. Even in double mutant clones that derepress Cyclin E and dE2F1, there is only a weak activation of S-phase in the posterior ([7,13,14]; our unpublished results). Together, these experiments confirm that the inhibitors of Cyclin E and dE2F1 are present and contribute to G1 arrest in the NPR. However, the relatively weak phenotype of their loss (at least in the posterior region) emphasises the presence of another inhibitory factor, which we have shown to be the dedicated inhibitor of Cyclin A, Rux.

Loss of Rux or overexpression of Cyclin A was sufficient to trigger ectopic S-phases, even when normal Dpp and Hh signals were present. Importantly, we have shown that *rux *expression does not require Dpp and Hh signalling, implying that control of the NPR by Cyclin A is distinct from that by Cyclin E and dE2F1. Since *rux *is expressed across the whole of the NPR territory, we envisage that it may provide a fixed barrier to S-phase entry, which must be overcome when cells legitimately enter S-phase in the second mitotic wave. Consistent with this view of Rux function, we observed no disturbance of the entry of cells into the NPR in *rux *mutants (not shown). This importance of Cyclin A further supports the growing body of evidence for an S-phase function for this more famously mitotic cyclin. This role also has been clearly identified in mammals where, although there is not a *rux *homologue, there are multiple controls that mediate Cyclin A inhibition in G1-phase.

## Conclusion

From a synthesis of our data with previous work, we propose the following outline of the full course of events that lead to the regulated synchronisation and arrest of proliferation in the NPR. As the morphogenetic furrow advances towards more anterior cells, they first experience the secreted Dpp and Hh signals that induce a coordinated entry into mitosis (the so-called first mitotic wave). After that, Dpp (but not Hh) causes them to downregulate Cyclin E levels and dE2F1 activity, thereby preventing S-phase entry and arresting them in G1. As the approaching furrow gets closer, and the cells mature in the posterior region of the NPR, other controls come into play. Most notably, Hh secreted from cells posterior to the furrow upregulates Dap expression, further ensuring the inactivity of Cyclin E. Hh also contributes at this stage to the repression of dE2F1. In addition to these signal dependent controls, Rux contributes to G1 maintenance by its inhibition of Cyclin A.

This overlapping set of mechanisms for ensuring that cells do not enter S-phase precociously appears strangely complex and redundant. Presumably this is a measure of the importance of this developmental regulation of proliferation. In the posterior region of the NPR, cells must prepare themselves for imminent changes as they begin either to differentiate as photoreceptors, or to re-enter S-phase for the terminal division of the second mitotic wave. These later decisions are precisely controlled by an overlapping and reiterated set of signals, including Hh and Dpp themselves [7,27,40]. The mechanism by which the same signals are used reiteratively to specify distinct fates could be confusing to a cell and is not fully understood by us, but is thought to rely on distinct transcriptional consequences of signals received by cells at different stages of their development. Perhaps the addition of Cyclin A repression and Dap expression in this important transitional region adds a failsafe mechanism to ensure the precise onset of retinal differentiation.

## Methods

### Genetic strains

The following alleles were used: *ato*^1^, *smo*^3^, *tkv*^*a*12^, *ci*^94^, *Mad*^12^, *rux*^8^, *UAS-ato, UAS-cycA*, *UAS-cycE*, *UAS-dE2F1*, *UAS-Dp*. All stocks are described in [41]. We used two reporter lines: *PCNA-GFP *[21] and *rux-lacZ *[15].

### Immunohistochemistry and BrdU labelling

Imaginal discs from third instar larvae were stained as described in [42]. The following antibodies were used. Guinea pig anti-Ato (1:100) (gift of H. Bellen); rabbit anti-Arm [43]; rabbit (Cappel) and mouse anti-β-Galactosidase; rabbit anti-GFP (kind gift of Rob Arkowitz); mouse anti-BrdU (1:10) (Becton Dickinson); rat anti-Ci (diluted 1:5) [44]; mouse anti-CycB (diluted 1:2); mouse anti-Dap (1:50) (gift of I. Hariharan); rabbit anti-phosphohistone H3 (1:100) (Upstate). Rat anti-E-cadherin (1:20), rat anti-Elav (1:100); and anti-CycB (1:5) were obtained from the Developmental Studies Hybridoma bank at the University of Iowa.

For BrdU labelling, imaginal disc were dissected and incubated with 200 μg/ml BrdU (Sigma) in PBS for 50 min. BrdU-labelled cells were detected as described previously [45].

### Confocal imaging and 3D reconstruction

For 3D reconstruction, eye imaginal discs were mounted between two strips of double side adhesive tape, using Fluoromount-G (Southern Biotech). Discs were analyzed with a BioRad Radiance 2100 laser scanning confocal. Z-series were projected for three-dimensional reconstruction using Volocity 2.5.1 software.

### Generation of mosaics

Mitotic clones were generated by FLP-mediated mitotic recombination [46]. Recombination was induced in second instar larvae (60 hr AEL) by a 120 min heat shock at 37°C. Mutant clones were marked in each case by the absence of β-gal antibody, Ci antibody, or GFP antibody staining.

Mutant clones were obtained of the following genotypes:

*y w hsF*; *Mad*^12 ^*FRT40A*/*M *[*ci*^+^] *FRT40A*; *ci*^94 ^[7]

*y w hsF*; *tkv*^*a*12 ^*FRT40A*/*M *[*ci*^+^] *FRT40A*; *ci*^94 ^[36]

*y w hsF*; *tkv*^*a*12 ^*FRT40A*/*ubi-GFP FRT40A*

*y w hsF*; *tkv*^*a*12 ^*FRT40A*/*arm-lacZ FRT40A; PCNA-GFP*

*y w hsF*; *smo*^3 ^*FRT40A*/*ubi-GFP FRT40A*

*y w hsF*; *smo*^3 ^*tkv*^*a*12 ^*FRT40A*/*ubi-GFP FRT40A*

*y w hsF*; *smo*^3 ^*tkv*^*a*12 ^*FRT40A*/*ubi-GFP FRT40A; rux-lacZ*/+

*y w hsF*; *smo*^3 ^*tkv*^*a*12 ^*FRT40A*/*arm-lacZ FRT40A; PCNA-GFP*/+

*y w hsF*; *smo*^3 ^*tkv*^*a*12 ^*FRT40A*/*arm-lacZ M(2)z FRT40A*

*rux*^8 ^*FRT18A*/*ubi-GFP FRT18A; hsF*/+

*rux*^8 ^*FRT18A*/*arm-lacZ FRT18A; hsF*/+; *PCNA-GFP*/+

*y w hsF*; *ato*^1 ^*FRT82B*/*arm-lacZ M(3)w124 FRT82B*

Clones of cells expressing Gal4 [28] were induced 48–72 hr after egg laying by 20 min heat shocks at 37°C. To generate clones of cells overexpressing *UAS-ato*, *UAS-cycA*, *UAS-cycE*, *UAS-dE2F1*, *UAS-Dp*, males carrying the different transgenes or combinations of them were crossed with *y w hsF*; *act-FRT y*^+ ^*FRT-Gal4 UAS-GFP*/*SM6a-TM6b Tb *females. In the case of the analysis of the *PCNA-GFP *expression in clones of *UAS-cycA*, males *y w hsF;UAS-cycA*; *PCNA-GFP *were crossed with *y hsF*; *ubx-FRT f *^+ ^*FRT-Gal4 UAS-lacZ*^*nls*^/*CyO *females. Overexpressing clones were marked by the presence of GFP or β-gal staining in each case.

## Authors' contributions

L.M.E. carried out all the experiments; L.M.E. and M.F. planned the study, and the wrote the manuscript together. Both authors read and approved the final manuscript.
